# Ecological Assessment of Two Species of Potamonautid Freshwater Crabs from the Eastern Highlands of Zimbabwe, with Implications for Their Conservation

**DOI:** 10.1371/journal.pone.0145923

**Published:** 2016-01-11

**Authors:** Tatenda Dalu, Mwazvita T. B. Sachikonye, Mhairi E. Alexander, Timothy Dube, William P. Froneman, Kwanele I. Manungo, Onias Bepe, Ryan J. Wasserman

**Affiliations:** 1 Zoology and Entomology, Rhodes University, Grahamstown, Eastern Cape, South Africa; 2 Environmental Science, Rhodes University, Grahamstown, Eastern Cape, South Africa; 3 Institute of Biomedical and Environmental Health Research, School of Science and Sport, University of the West of Scotland, Paisley, Scotland; 4 School of Agriculture, Earth and Environmental Sciences, University of KwaZulu-Natal, Pietermaritzburg, KwaZulu Natal, South Africa; 5 Chimanimani National Park, Chimanimani, Manicaland, Zimbabwe; 6 Nyanga National Park, Nyanga, Manicaland, Zimbabwe; 7 South Africa Institute for Aquatic Biodiversity, Grahamstown, Eastern Cape, South Africa; National University of Singapore, SINGAPORE

## Abstract

The spatial ecology of freshwater crabs and their conservation status is largely understudied in Africa. An ecological assessment was conducted at 104 localities in 51 rivers and/or streams in the Eastern Highlands of Zimbabwe whereby the distribution and abundances of freshwater crab species were mapped and the possible drivers of the observed trends in population structure explored. In addition, information on crab utilisation as a food resource by local communities was assessed via face to face interviews across the region. Finally, the conservation status of each species was assessed using the IUCN Red List criteria. Only two crab species *Potamonautes mutareensis* and *Potamonautes unispinus* were recorded within the region of study. *Potamonautes mutareensis* was largely restricted to less impacted environments in the high mountainous river system, whereas *P*. *unispinus* was found in low laying areas. In stretches of river where both species were found to co-occur, the species were never sampled from the same site, with *P*. *mutareensis* occurring in shallower, faster flowing environments and *P*. *unispinus* in deeper, slow flowing sites. Interview results revealed that the local communities, particularly in the southern part of the Eastern Highlands around the Chipinge area, had a considerable level of utilisation (55% of households) on the harvesting of crabs for household consumption during the non-agricultural season (May to September). Results from the IUCN Red List assessment indicate that both species should be considered as “Least Concern”. Threats to freshwater crabs in the Eastern Highlands, however, include widespread anthropogenic impacts such as habitat destruction associated with gold and diamond mining, inorganic and organic pollution and possibly exploitation for human consumption. The current study provides important information and insight towards the possible development of a freshwater crab conservation action plan within the region.

## Introduction

The ecological role of freshwater crabs is often overlooked in aquatic ecosystems, despite their wide distribution in tropical and warm temperate zones [1]. Crabs are important detritivores, reducing the leaf litter and organic debris particle size, thus presenting a source of nutrition for other macroinvertebrate fauna and enabling microbial activity [1–4]. Moreover, crabs are important prey items, forming the intermediary trophic link between detritus and predacious animals such as fish, otters, mongoose and birds [4–7]. Given their contribution to invertebrate biomass and their importance as prey items, freshwater crabs constitute an integral component of food webs in freshwater aquatic ecosystems [1, 4]. In addition, they are of considerable economic importance in parts of Africa as they can form a significant part of the diet for people in selected rural areas [4, 8, 9].

As of the year 2014, the freshwater crab taxa of Africa are considered to be comprised of ~126 species, which are currently assigned to eleven genera and four families [4]. On the continent, freshwater crabs are present in almost all freshwater habitats, from mountain streams to large lowland rivers and small water bodies, often constituting the largest invertebrate biomass [3, 10–16]. Owing to their generally limited dispersal abilities, most true freshwater taxa are endemic to certain ecoregions [4, 17–19]. In the southern African region, some twenty-five species of freshwater crabs belonging to the genus *Potamonautes* MacLeay 1838 are known to occur and there is a strong likelihood that the number of species will increase as taxonomy progresses and exploration continues in this relatively understudied field [13, 15]. *Potamonautes* belongs to the Potamonautidae, a family endemic to the Afro-tropical region [4, 10, 17].

The biogeographic affinity of sub-Saharan African freshwater crabs is relatively unknown, particularly with respect to the genus *Potamonautes* [4]. Understanding the biogeography of freshwater crab taxa can reveal possible connectivity between habitats, especially in river systems. The species composition of the freshwater crab fauna and their ecological role in Zimbabwean freshwater systems has received little attention to date [5–7, 19, 20]. The present study, therefore, seeks to investigate the distribution patterns and relative abundances of freshwater crabs belonging to the genus *Potamonautes*, in the Eastern Highlands of Zimbabwe.

Based on evidence of the existence of endemic *Potamonautes mutareensis* in the Penhalonga-Juliasdale area [19] and other more widespread *Potamonautes* spp., a comprehensive survey of the Eastern Highlands region was conducted, comprising two distinct components. Firstly, an ecological assessment was completed in the Eastern Highlands of Zimbabwe, whereby the distribution and abundances of freshwater crab species were mapped and the possible drivers of the observed trends in the populations explored. Secondly, information on the utilisation of crabs as a food resource by local communities was assessed in select villages across the study area, through face to face interviews. Finally, we present an outline of a case study in a protected area highlighting the efficacy of locally directed conservation efforts. It is hoped that findings of this research will help identify priority areas for protection of the different crab species and highlight any potential conflicts with possible future conservation priorities. Sound knowledge of species ecology, population size and distribution as well as an understanding of the social component that interacts with the environment are fundamental requirements for the design and implementation of effective conservation and management strategy. The purpose of this study was, therefore, to generate scientific data and provide information that will guide policy makers on how to protect and conserve these “*endemic*” and other non-endemic crab species of the Eastern Highlands of Zimbabwe. We anticipate that this study could aid conservation measures through providing information on population size, extent of occurrence (EOO), area of occupancy (AOO) and existing threats.

## Methods

The collection and ethics permits were approved by National Parks and Wildlife Management of Zimbabwe (NPWM; permit no. 23(1)(C)(II)01/2015) and Rhodes University Ethics Committee, permit no. CRO 17/14CR.

### Study area

**Fig 1:**
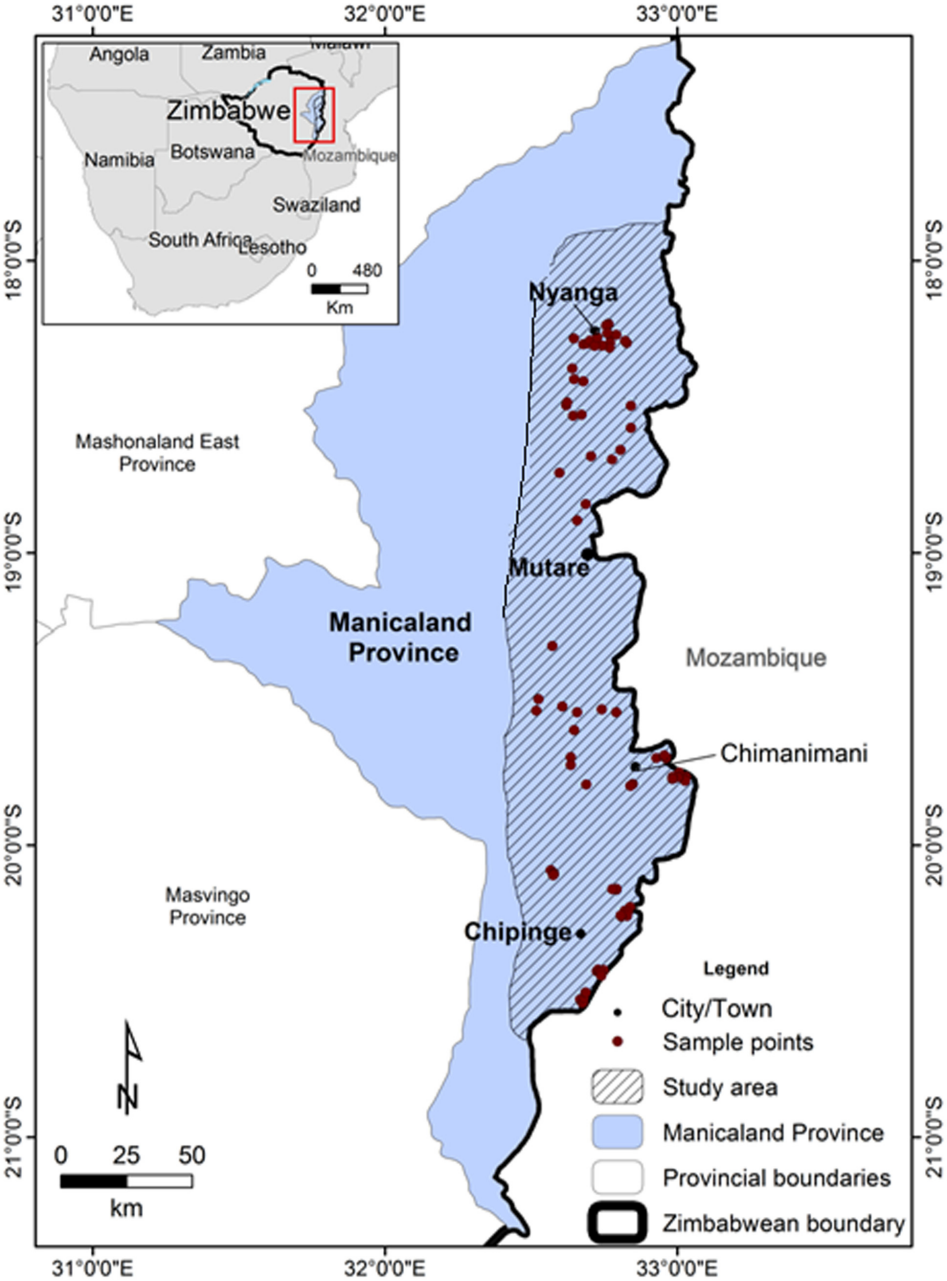
Location of the sampling location in the Eastern Highlands, Zimbabwe.

The Eastern Highlands, as it is known in Zimbabwe, is a narrow mountain belt (~450 km long north—south), located on the eastern Zimbabwe—western Mozambique border (Fig 1). The northern most region is known as the Nyanga Highlands, the central region as the Vumba Highlands and Chimanimani Mountains, with the Chipinge Highlands situated in the southern region [19, 21]. The mountain range comprises a complex mosaic of vegetation types including forests, woodlands, and grasslands [22, 23], with the fauna showing affinities to these respective habitat types and includes several animals/plants endemic to the region [21–24]. The hilly and generally inaccessible nature of these mountains has preserved much of the native flora and fauna [21].

The annual rainfall in the ecoregion is highly variable, ranging from 741–2997 mm, falling during the austral summer months (November to April) [22, 25]. The mean annual temperatures range from a minimum of 9–12°C to a maximum of 25–28°C [21, 23, 26]. Freshwater crabs and physico-chemical variables were sampled and measured from 101 localities in 51 rivers and/or streams in the Eastern Highlands, Zimbabwe during the summer (January 2015; Fig 1). Much of the sampling in the south of the Eastern Highlands was conducted on the Tanganda Tea Estates, namely Jersey, New Year’s Gift, Ratelshoek, Tingamira and Zona (Fig 1).

### Field sampling

Physico-chemical variables (i.e. conductivity, dissolved oxygen [DO], pH, total dissolved solids [TDS], salinity and water temperature) were recorded at each site using a portable handheld multi-parameter PCTestr 35 (Eutech/Oakton Instruments, Singapore) and DO meter (Thermo Fisher Scientific, USA). Water samples (150 mL) were collected from each site for nutrient analysis (i.e. ammonium and phosphates) and placed on ice in a cooler box. The water samples were analysed within 14 hours of collection using the Hanna Instruments phosphate high range checker (HI717) and ammonia test kit for freshwater (HI3824; Hanna Instruments, Rhode Island). The portable meter phosphate range was 0–30 mg L^-1^ and resolution 0.1 mg L^-1^, whereas ammonia had a range of 0–2.5 mg L^-1^ and resolution 0.1 mg L^-1^. Habitat and basin characteristics such as channel width, canopy cover, litter composition (% cover), macrophyte cover, substratum embeddedness and water depth at each site were also recorded according to [27]. Sites were also visually assessed for obvious signs of habitat degradation associated with anthropogenic activities (i.e. illegal gold panning) and pollution.

Mixed method samples were used for the collection of freshwater crabs whereby hand sampling, baited ox-heart lines [19] and nylon hand nets (mesh size 500 μm, dimension 30 × 30 cm) were employed, cumulatively comprising a single sample for a particular site. At each sampling site, freshwater crabs were sampled by submerging the sampling net and sweeping through a demarcated 10 m transect in a zig-zag fashion, while actively searching in the macrophytes and over turning the rock substratum [1]. Baited ox-heart lines left in the water for a period of 30 min at five different locations within the 10 m transect, with all crabs observed within the 2 m perimeter being counted. After counting the number of crabs at each location, a subsample (n = 3) of the representative species was collected, after which they were killed by freezing for 24 hrs and then transferred to 95% ethanol for further verification using DNA analysis by the Evolutionary Genomics Group in the Department of Botany and Zoology at Stellenbosch University, South Africa. Freshwater crabs were identified using keys by Phiri and Daniels [19].

### Data analysis

The effects of the environmental variables on the abundance of each crab species were analysed using generalised linear mixed models (GLMMs) with a Poisson error distribution and logit link using Laplace approximation. Region (north, central or south) was incorporated as a random effect. We initially used the variance inflation factor (VIF) to test for multi-collinearity among all variables and deleted those factors that returned VIF values >3 ([28]; Table 1). Pearson correlations were also examined within each GLMM to verify that variables in the model were not highly collinear. GLMMs were fitted and unimportant variables were removed via stepwise deletion: only variables significant at the 5% level were retained in the final model. GLMMs were fitted in the ‘lme4’ package [29] and all analyses were conducted in R [30]. An independent sample t-test was used to determine the difference in the number of crabs found in impacted (where pollution was noted i.e. sewage discharge, gold panning) and non-impacted areas (i.e. relatively pristine areas mostly in protected areas) using SPSS version 16.0 [31].

**Table 1:** Environmental variables included in GLMM analyses.

Variable	Description
Altitude	Elevation, above sea level (m)
Dissolved oxygen	The level of oxygen that is dissolved in the water (mg L^-1^)
pH	Numeric scale used acidity or alkalinity of the water
Conductivity	Capacity of water to conduct electricity (ppm)
Temperature	Physical property expressing how cold or hot water is (°C)
Water depth	The maximum water depth (m) were the crabs were found
Channel width	Width of the river with water flowing (m)
Phosphates	Concentration of phosphate ions (mg L^-1^) in water column
Clay	Proportion (%) of clay substratum per 10 m transect
Sand	Proportion (%) of sand substratum per 10 m transect
Bedrock	Proportion (%) of bedrock substratum per 10 m transect
Macrophytes	Proportion (%) of macrophyte cover per 10 m transect
Detritus	Proportion (%) of detritus cover per 10 m transect

Geographical positions of study locations were used to produce distribution maps based on the estimated abundances of freshwater crabs. The spatial distribution of the two crab species *(i*.*e*. *Potamonautes mutareensis* and *Potamonautes unispinus)* sampled within the study area was modelled in a GIS environment based on the species dataset collected during field-surveys using freely obtained images from the United States Geological Survey (http://glovis.usgs.gov/). The dataset was imported into ArcGIS 10.2 software [32] and then processed to show the distribution patterns across the entire study area. The distribution map was implemented using the graduated symbolization interface in ArcGIS [32]. This interface uses the quantitative values or species population data gathered during field surveys to sub-divide the dataset into distinct classes. Within each class, all the features were then highlighted with the symbol and symbol size.

Each of the two freshwater crab species found in the Eastern Highlands region was evaluated against the IUCN (2003) Red List criteria to assess their risk of extinction [4, 13, 33] for the study region. The conservation assessment was based on extent of occurrence (EOO), area of occupancy (AOO), crab abundances and possible threats. Threats for a particular species were based on the presence of potential anthropogenic impacts such as habitat destruction associated with mining impacts (gold panning) and pollution (toxic or sewage discharge).

The human participants’ ethics clearance was approved by Department of Zoology and Entomology, Rhodes University Ethics Committee. No written consent was acquired from participants of the survey, as most community members were reluctant to have their names recorded due to the prevailing political situation in Zimbabwe. This issue was raised to the Ethics Committee before the study began and the necessary use of verbal consent was therefore approved by the ethics committee. Consent was therefore noted in the interview document to show that the person agreed to be part of the study. All participants gave verbal informed consent and were thoroughly debriefed. Verbal consent was considered to be sufficient, since it was ensured that data were stored and analysed anonymously. The individuals' verbal consent was obtained after reading the instructions to the candidate.

Informal open-ended face to face interviews (n = 38: north– 10, central– 12, south– 16) were conducted randomly within the local communities where freshwater crabs were collected to assess attitudes towards their conservation. The researchers first requested verbal permission to carry out the interview with the participating individuals followed by an explanation on the nature of the study. The participants were in no way coerced into participating and were informed of their right to refuse to be involved, or to withdraw at any point during the interview process. The researchers ensured that participation into this study was voluntary and the prospective participants were fully informed about the procedures and the associated risks involved in research. This method was expected to produce useful information for the implementation of any local action plan for freshwater crab conservation. The interview questions probed responses of knowledge of conservation ideals to 1.) Establish cognitive barriers and enablers towards conservation, and 2.) Establish whether or not the conservation of freshwater crab species was important to the community (see S1 Text). This information was used to assess for conflicts of interest between economic activities and potential conservation efforts. Differences in habitat threats e.g. illegal mining (i.e. gold panning), organic pollution, agriculture, crab harvesting across three regions: northern, central and southern highlands were tested for significance using a chi-squared test of independence in Microsoft Excel 2007. To highlight usage of crabs and mining as a threat to crab abundances, we linked the degree of household usage of crabs (through interviews) and mining activity to the actual crab abundances found (through their sampling efforts) using Pearson correlations in SPSS version 16.

## Results

### Habitat characteristics

Of the 16 environmental variables that were measured, ammonia, TDS and pebble substrates were found to be collinear with other variables so were removed from further analyses. Of the thirteen remaining variables, the abundance of both *Potamonautes unispinus* and *Potamonautes mutareensis* across the three regions was found to be significantly associated with conductivity, clay and macrophyte presence (Table 2). *Potamonautes unispinus* was also associated with altitude, pH, temperature, channel width and phosphorus whilst *P*. *matureensis* was associated with dissolved oxygen and water depth (Table 2). Variation in the mean environmental variables recorded in the study locations where the two crab species were found in the Eastern Highlands are highlighted in S1 Table.

**Table 2:** GLMM estimates for relationship between abundance of both *Potamonautes unispinus* and *Potamonautes mutareensis* and predictor environmental variables that were found to be significant via stepwise deletion of non-significant variables.

Explanatory variable	Estimate (±SE)	z	*p*
*P*. *unispinus*			
Channel width	-0.054±0.011	-4.72	<0.001
Clay	-0.726±0.274	-2.65	0.008
Conductivity	-0.009±0.002	-6.05	<0.001
Altitude	-0.001±0.0003	-4.71	<0.001
Macrophyte cover	-1.573±0.236	-6.67	<0.001
pH	-0.343±0.072	-4.79	<0.001
Phosphates	-0.161±0.048	-3.39	<0.001
Temperature	0.064±0.024	2.73	0.006
*P*. *mutareensis*			
Bedrock	-0.436±0.178	-2.45	0.014
Clay	0.787±0.187	4.21	<0.001
Conductivity	-0.006±0.002	-3.78	<0.001
Dissolved oxygen	0.045±0.443	3.66	<0.001
Macrophyte cover	0.741±0.212	3.49	<0.001
Water depth	0.237±0.094	2.52	0.011
Temperature	-0.036±0.018	-1.98	0.047

### Distribution patterns

**Fig 2:**
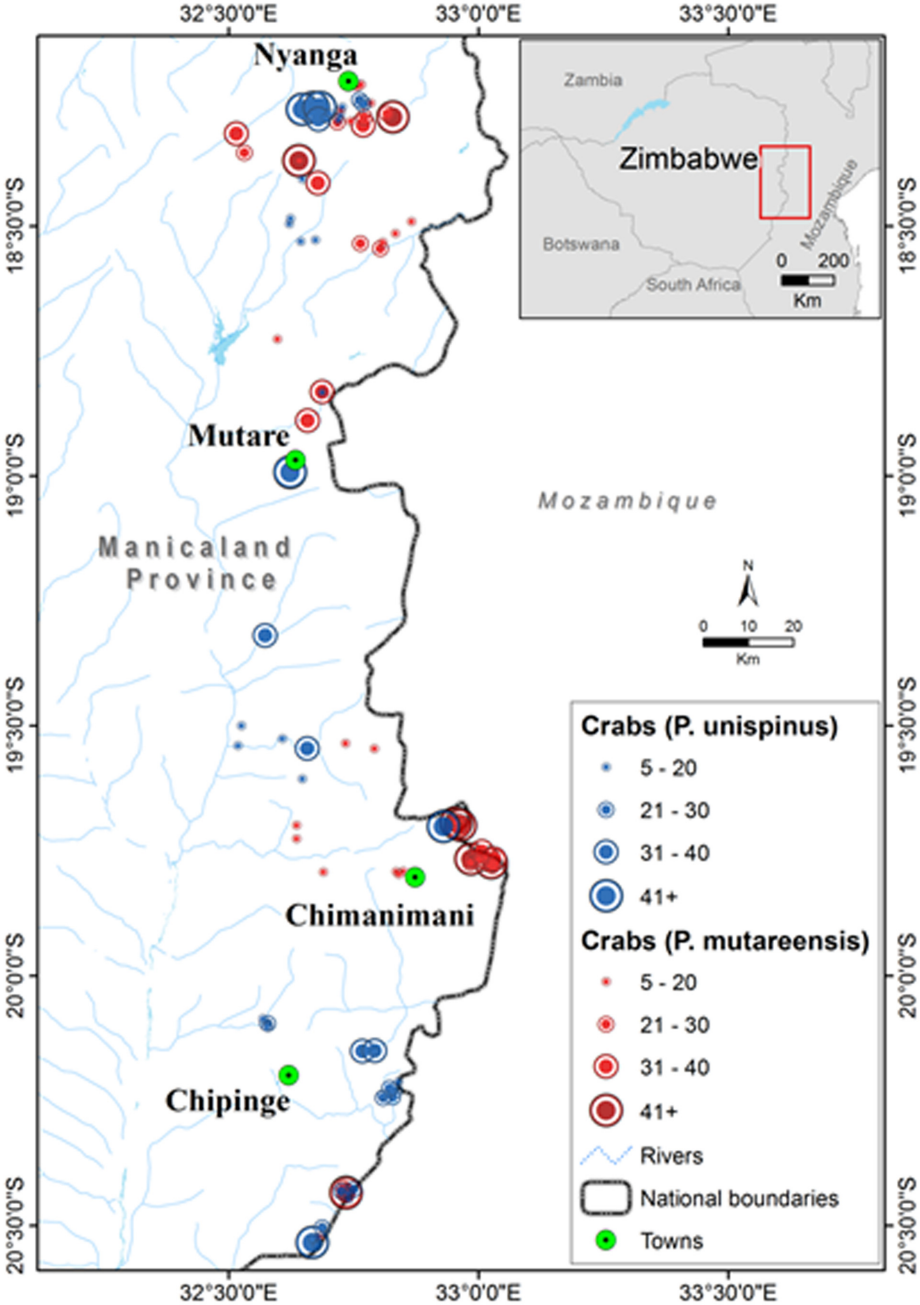
Distribution map of *Potamonautes mutareensis* and *Potamonautes unispinus* in the Eastern Highlands of Zimbabwe, showing locations of major town.

Of the four known freshwater crab species found in Zimbabwe belonging to the Potamonautidae family, two have been recorded in the Eastern Highlands of Zimbabwe (S2 Table). In the present study, however, *Potamonautes perlatus* which was thought to be restricted to the edge of the Eastern Highlands, north of Nyanga and to the northern parts of Zambezi river system (Kairezi River system, Northern Zimbabwe) [5–7], may have been mis-identified, as this species is now considered to be endemic to South Africa [13, 16]. Throughout the sampled areas of the Eastern Highlands, the only stretches of river where *P*. *mutareensis* and *P*. *unispinus* were found to co-occur was in the Chimanimani, Nyanga and Zona regions which was likely due to the increased habitat heterogeneity in these areas (Dalu *pers*. *obs*.). In the region where the two species were sympatric, a high degree of habitat partitioning was observed at the finer scale. *Potamonautes unispinus* was found mostly in low lying areas and attained their highest numbers (n = 80) in the sewage polluted river systems of Mutare (Fig 2). By contrast, *P*. *mutareensis* was mostly associated with rivers found in the high mountainous regions of the Eastern Highlands, with high abundances (n = 63) being recorded in the Chimanimani Mountains, in a protected national park (Fig 2). While these two species overlapped at the stream scale, they were never found to co-occur within a given site with *P*. *unispinus* sampled from deeper pools in wide sections of the river and *P*. *mutareensis* sampled from riffles and narrow river channel environments, suggesting a degree of microhabitat partitioning within areas of overlap. *Potamonautes unispinus* persist in organic (sewage) polluted waters in Mutare suggesting that it is tolerant to pollution, however, the organic pollution tolerances for *P*. *unispinus* and *P*. *mutareensis* are currently unknown.

### Threats, impacts and conservation

**Fig 3:**
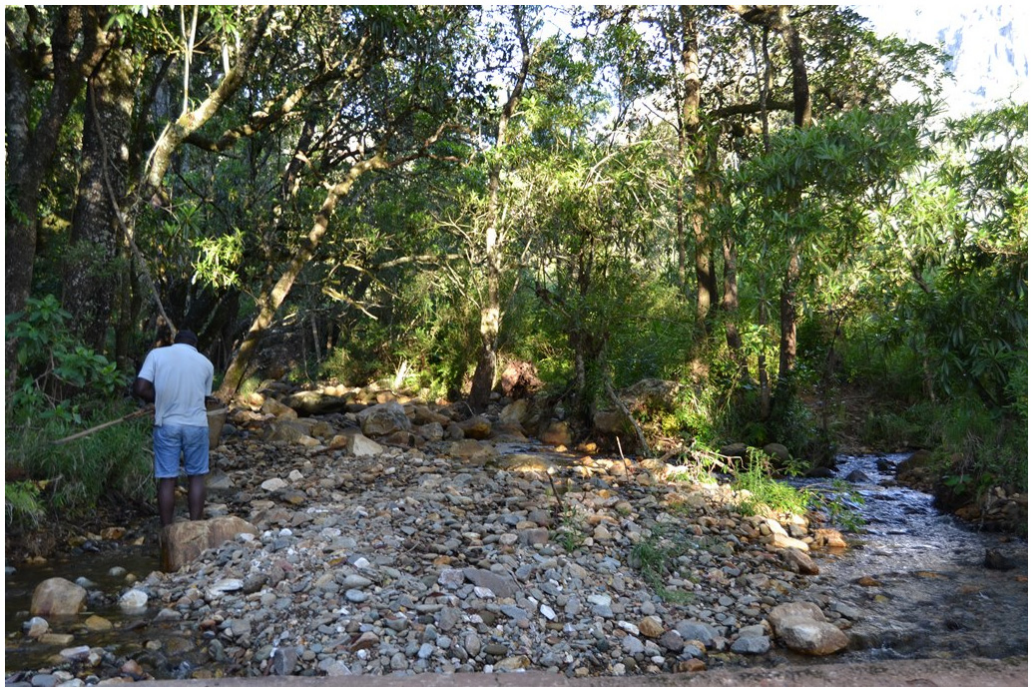
Impact of gold panning on aquatic ecosystem habitats along Bindu River, Chimanimani. Note the transferal of rocks and gravel from the stream bed on to the margins where it is worked for gold. This activity results in loss of habitat and alters downstream water quality.

**Fig 4:**
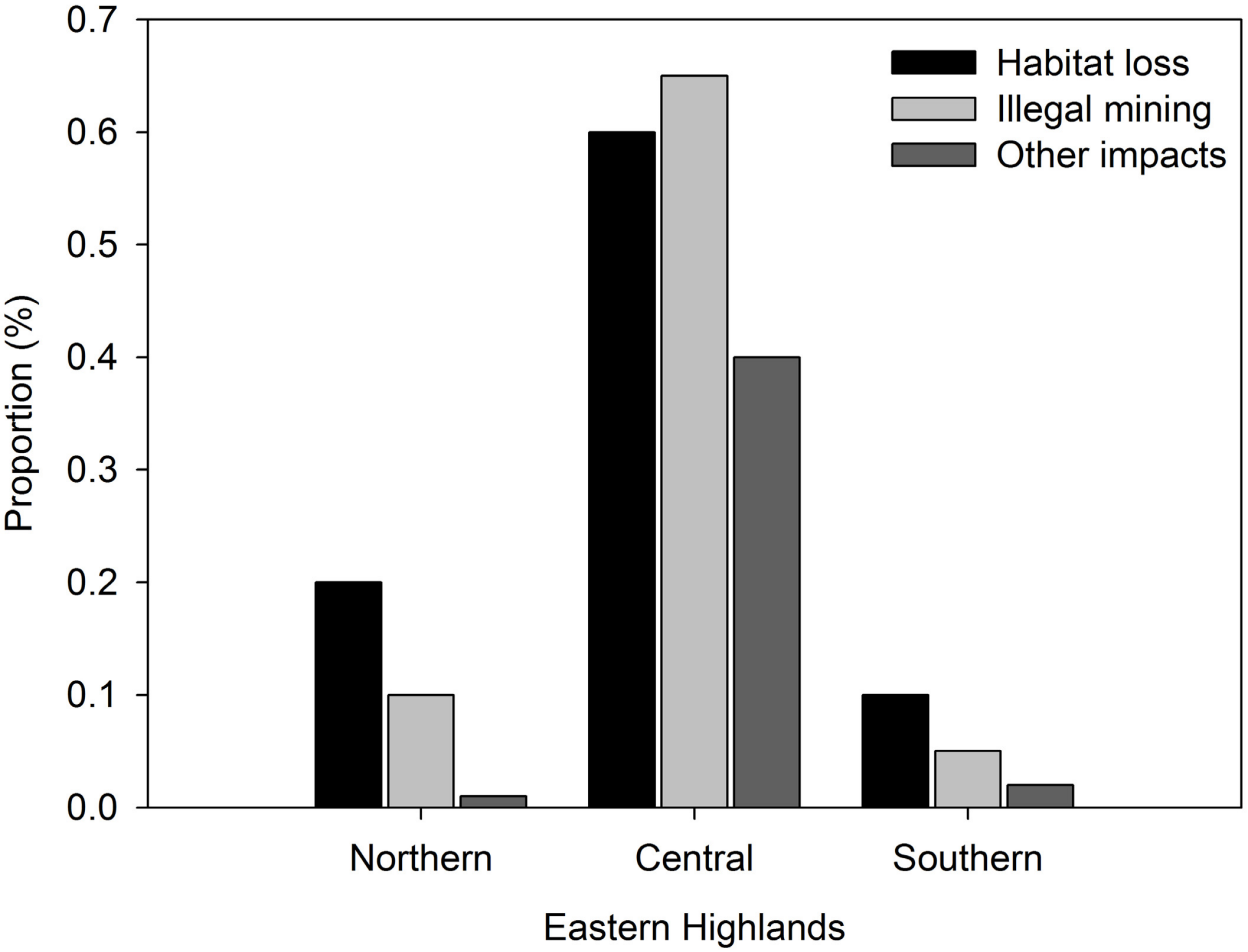
Anthropogenic impact effects on freshwater crab habitats observed in the Eastern Highlands of Zimbabwe.

*Potamonautes mutareensis* (EOO = ~52000 km^2^, AOO = ~520 km^2^, localities = 59) and *P*. *unispinus* (EOO = >310000 km^2^, AOO = >3100 km^2^, localities = 88) were both found to be of least concern due to their widespread distribution and at times, high abundances (Fig 2; S2 Table). Threats to freshwater crabs in the Eastern Highlands, however, include widespread anthropogenic impacts such as habitat destruction, inorganic and organic pollution (Figs 3 and 4) and possibly exploitation for human consumption. One particular activity of potential conservation concern is illegal gold panning in the streams (see Figs 3 and 4) and diamond mining particularly in the Chimanimani (55%), Chiadzwa and Marange (70%) and Penhalonga (65%) sites. We observed significantly lower crab populations in impacted areas (t = 2.837, *p* = 0.031). No significant differences were observed using Kruskal-Wallis (H = 1.120, df = 2, *p* = 0.571) for crab abundances in national parks, rehabilitated areas and other areas (i.e. areas outside national parks and rehabilitated areas). Negative correlations for crab abundances with crab utilisation (*r* = -0.189, *p* = 0.879) and mining (*r* = -0.945, *p* = 0.212) were observed for the three sampling regions, suggesting that these activities may have an effect on crab abundances.

The interviewed households in these areas were found to greatly depend on this economic activity (χ^2^ = 5.835, *p* = 0.028); respondents indicated that they would continue with gold panning in order to sustain their livelihoods. The interview results revealed that the communities in the southern part of the Eastern Highlands, around the Chipinge area, had a considerate dependence (56.3%) on the harvesting crabs for household consumption during the non-agricultural season extending from May to early September, while in the central region, ~10% of the community ate crabs. Significant differences were observed in freshwater crab harvesting activities across the northern, central and southern highlands (χ^2^ = 9.433, *p* = 0.009), with more crabs being utilised in the central region compared to the north and south regions. Most of the interviewees highlighted that they have been harvesting crabs since childhood and the reason for dependence on crabs during the non-agricultural season was to supplement their diet. The communities suggested that they only harvested big crabs and this was seen to adhere to conservation ideals, as harvesting took into consideration the crabs breeding patterns, with small or juveniles crabs being left to grow. Responses made to the informal open-ended face to face interview questions with the local people in the north, central and south of the Eastern Highlands of Zimbabwe are highlighted in Table 3.

**Table 3:** Responses made to the informal open-ended face to face interview questions (see Appendix 1 for questions) with the local people in the north, central and south of the Eastern Highlands of Zimbabwe.

Responses to questions	North	Central	South	Chi-square test
**Question 1**				
Not aware	70	41.7		**14.782 (0.001)**
Aware	30	58.3	100	
**Question 2**				
Provisional	10	16.7	42.7	4.457 (0.108)
Do not know	90	83.3	56.3	
**Question 3**				
Yes		8.3	56.3	**9.433 (0.009)**
No	100	91.7	42.7	
**Question 4**				
Occasional			6.3	1.412 (0.494)
No	100	100	93.7	
**Question 5**				
> 10 years			66.7	1.669 (0.196)
< 10 years		100	33.3	
**Question 6**				
Less a month per year			6.3	0.067 (0.796)
Hardly or not at all		100	93.7	
**Question 7**				
Between May and September		100	77.8	0.277 (0.598)
In July-August			22.2	
**Question 8**				
The crabs are bigger and abundant			44.4	0.740 (0.390)
I am not busy		100	55.6	
**Question 9**				
Not really, it’s different every year		100	77.8	0.277 (0.598)
No			22.2	
**Question 10**				
Amongst the detritus and rocks			55.6	1.113 (0.291)
Shallow, vegetated pools		100	44.4	
**Question 11**				
They have more food		100	11.1	**4.447 (0.035)**
It's their habitat			33.3	
I don't know			55.6	
**Question 12**				
I only take the big ones		100	22.2	2.595 (0.107)
I only harvest when I don't have food			55.6	
None			22.2	
**Question 13**				
Yes		100	22.2	2.595 (0.107)
Maybe			11.1	
I don't know			66.7	
**Question 14**				
Yes		100	66.7	0.476 (0.490)
No			33.3	
**Question 15**				
Subsistence farming	50	33.3	43.8	6.271 (0.394)
Gold panning		41.7	12.5	
Seasonal farm labourer	20	16.7	31.3	
other	30	8.3	12.5	

## Discussion

A comprehensive survey of the Eastern Highlands region yielded two freshwater species, *Potamonautes mutareensis* and *Potamonautes unispinus* which were spatially separated by physico-chemical variables and altitude/elevation. Findings of this research could potentially assist in the identification of future priority areas for protection of the two crab species. The study revealed that population numbers for both species were significantly higher in the protected areas i.e. Chimanimani and Nyanga National Parks and Tanganda Tea Estates (Fig 2). High levels of habitat destruction, such as illegal gold panning and pollution were observed outside these protected areas, and, as such, these habitat alterations were likely having an effect on population levels of both species, particularly the more sensitive *P*. *mutareensis*. There is, however, some evidence to suggest that *P*. *mutareensis* extends its distribution into Mozambique. Thus, the present study provides more information on the population size and distribution of *Potamonautes* spp. within the Eastern Highlands, as well as an understanding of species’ habitat ecology, conservation status, and threats, with appropriate measures to mitigate them being identified.

Understanding habitat characteristics of organisms such as crabs, may better place conservationists in protection of a species. The present study has provided baseline ecological data which could be useful for future monitoring and management of freshwater crabs in the Eastern Highlands area. The study shows that *P*. *mutareensis* and *P*. *unispinus* seemed to respond to certain environmental variables. Altitude, substrate characteristics (i.e. clay, bedrock), hydromorphology (i.e. channel width and water depth), macrophyte cover and water chemistry (i.e. pH, temperature and conductivity) were all found to be potentially influential predictors of crab species distribution.

Several studies such as Dallas [34] and Chakona et al. [35] have highlighted that habitat complexity is an important factor structuring invertebrate assemblages in river systems. In our study, high abundances of *P*. *mutareensis* were associated with high macrophyte cover, dissolved oxygen, water depth, clay and low temperature. This might explain the existence of *P*. *mutareensis* at high altitude with high habitat complexity as habitat complexity has been highlighted to increase with water depth in many undisturbed river systems [35, 36]. Boyero and Bailey [37] also highlighted that complex and stable habitat provides refuge from high water velocity and provides food. In contrast, high abundances of *P*. *unispinus* were associated with low altitude, pH, macrophyte cover and high temperatures. Although *P*. *unispinus*, was found in high abundances in anthropogenic impacted rivers (i.e. mining and organic pollution), GLMM analysis found a negative relationship between phosphates and *P*. *unispinus*. The phosphate range where the *P*. *unispinus* was found in impacted areas was 1.2–4.5 mg L^-1^ compared to 0.1–2.1 mg L^-1^ in low impacted areas. This suggests that the species might have a limit or tolerance for anthropogenic impact up to a certain level and this might prove to be critical to the species, if they continue to be affected by anthropogenic impacts.

### Case study of Tanganda Tea Estates: Conservation in action

**Fig 5:**
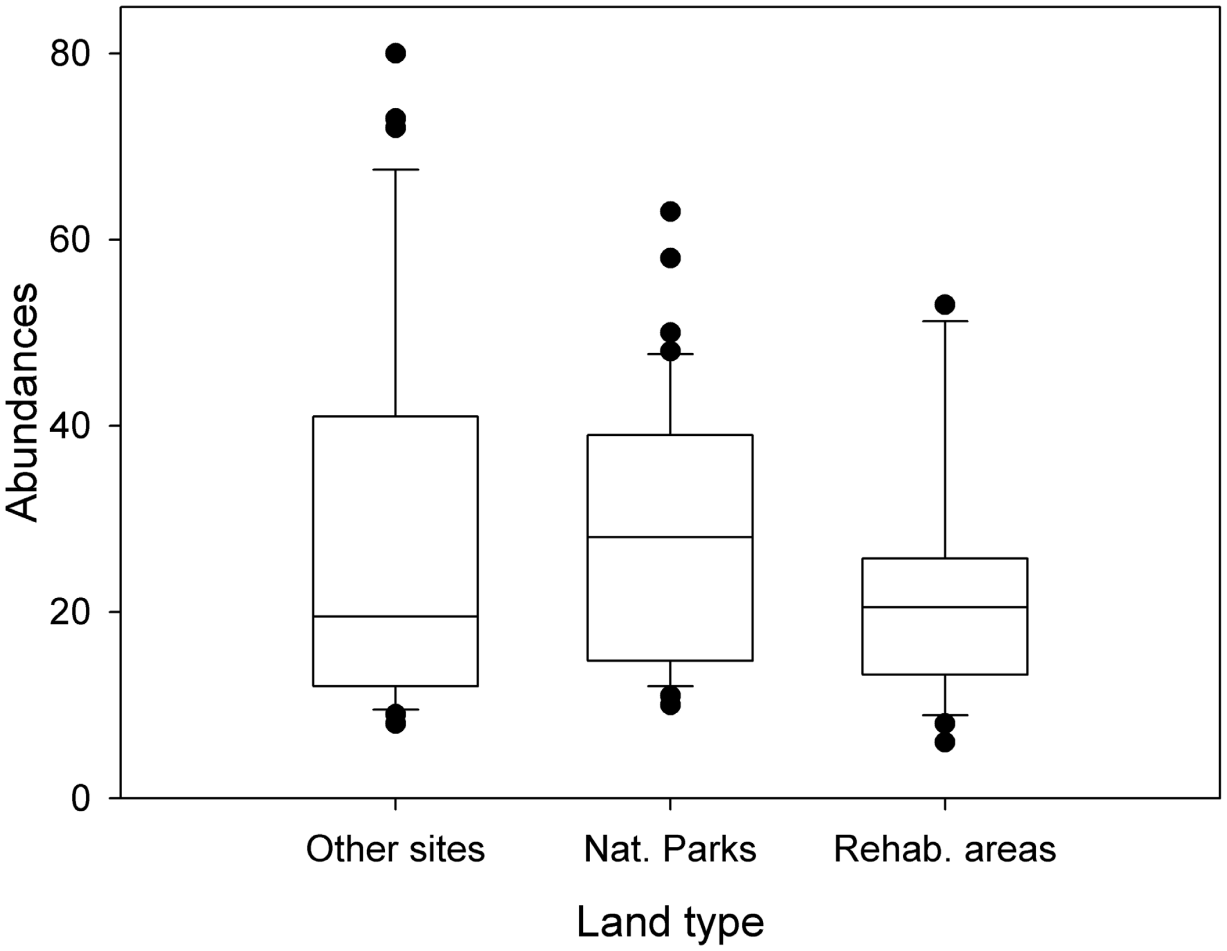
Differences in crab abundances in the three land types of the Eastern Highlands, Zimbabwe. Abbreviations: Nat. Parks—National Parks; Rehab. Areas—Rehabilitated areas (i.e. tea estates); Other sites—sites outside national parks and rehabilitated areas.

Freshwater crab conservation relies on the preservation of patches of natural forest large enough to maintain good water quality and leaf litter for food since many species are sensitive to anthropogenic impacts [4, 13]. Tanganda Tea Estates forms part of a conservation initiative known as “Rainforest Alliance”, which advocates for the protection, preservation and sustainable use of natural ecosystems (www.rainforest-alliance.org/). In line with this, there has recently been extensive rehabilitation of natural forests and wetlands on select properties, namely the New Year’s Gift, Jersey, Ratelshoek, Tingamira and Zona tea estates. As a result of this initiative, the two crab species *P*. *mutareensis* and *P*. *unispinus* indirectly receive a measure of protection within these estates. Indeed the crab population of *P*. *mutareensis* was found to be slightly higher when compared to impacted areas i.e. sites outside national parks and the tea estates (Fig 5). National parks and Tanganda Estates management efforts are to be commended in conservation of natural forests/wetlands, as their efforts may contribute to the conservation of freshwater crabs and act as a source of refugee for the crab populations (Figs 2 and 5). Indeed, the conservation of small habitat fragments has been successful for crabs in many other parts of the world such as the conservation of the *Johora singaporensis*, an endemic potamid in the Bukit Timah Nature Reserve, Singapore [4, 38, 39].

### Conservation actions, challenges and recommendations

The IUCN (2003) Red List criteria results for the Eastern Highlands found the two species, *P*. *mutareensis* and *P*. *unispinus* to be of “Least Concern” (LC) as previously highlighted by regional and global scale assessments [4, 14, 33]. This is in contrast to several other aquatic taxa such as fish [40, 41] and amphibians (R. Hopkins, National Museums and Monuments of Zimbabwe, *pers*. *comm*.) within the same region which are currently considered as “Threatened”. *Potamonautes mutareensis*, however, has a fairly narrow distribution (~450 km) and is largely restricted to high altitudes. As such, it is likely that temperature may be an important factor driving its distribution, given that the higher altitudes are generally cooler, in an otherwise arid tropical part of the continent [42, 43]. While this may render *P*. *mutareensis* vulnerable to warming predicted by certain climate change prediction models [43], the species is likely more at risk from anthropogenic impacts such as gold panning. Although 55% of the local respondents indicated that they do exploit crabs as a food resource, this activity is limited to a very short period. In this way the communities in the central and southern part of the Eastern Highlands are unintentionally conforming to certain conservation ideals as crabs are only harvested during the peak breeding season (i.e. summer) and seem to be selecting only large crabs (Table 3). It would thus appear that the exploitation of crabs as a food resource poses a limited threat to the crabs as taking adults and leaving the rest of the population would allow normal recruitment to proceed. However, local respondents do not throw back ovigerous adult females or adult females carrying hatchlings hence these could have a considerable negative impact on crab populations over time. In addition, illegal mining in the region, mostly around the Chimanimani area was found to be a significant intermediate threat as it involves the large scale modification/destruction of natural habitats through the moving and removal of vast portions of gravel and rock, significantly affecting the crab abundances. In addition to the destruction of microhabitats in the streams, this activity is associated with increases in downstream turbidity which has been shown to have negative effects on crustaceans, macroinvertebrates and vertebrates [44–47]. Despite these threats, the current *P*. *mutareensis* population levels appear to be healthy in protected areas where mining is prohibited, minimal and/or non-existent.

Polices of the Zimbabwean government have contributed to a shrinking in the economy which has been associated with the increased exploitation of natural resources by impoverished rural communities. Moreover, the formal mining sector is under the auspices of Russian, Chinese and local operators many of whom have little regard for conservation [48, 49]. Social changes due to economic collapse have also impacted the environment as it lead to illegal mining causing habitat destruction and the ecosystems were further impacted by the discovery of diamonds and gold in the Eastern Highlands. Regrettably, these activities in the fragile Eastern Highlands represent major threats to aquatic and terrestrial fauna and flora. In order to prevent future habitat loss and conservation of the freshwater crabs, we advocate a four pronged approach; research, education, policy and management. Using research, we anticipate that the effects of anthropogenic impacts on freshwater crab populations can be quantified especially for *P*. *mutareensis* while incorporating an adaptive management approach whereby restoration methods that improve habitat and population sizes are identified. Through education, we advocate for the development and implementation of a conservation curriculum for primary, secondary and high schools. Outreach programs should be carried out to educate the local communities on conservation and management. On policy, we campaign that policy makers (i.e. legislators—members of parliament and senators) be educated on anthropogenic impacts of industry, land use, and agriculture on the local environment, and the long-term consequences for human health and local economies so that they can come up with policies to better conserve the environment and further ensure that policies are implemented and adhered to. Finally, management will involve all the stakeholders from the local communities, media, scientists and policy makers to adhere to environmental management guidelines outlined by conservation managers, so as to minimize future habitat loss.

Globally, biodiversity loss is of major concern and conventional mitigation approaches are often rather unsuccessful as action occurs at the species level and only when a species is in under threat of extinction [50]. To provide a solution to this problem, the Convention for Biological Diversity [51] has advocated for the promotion of conservation of species and ecosystems within protected and well managed areas i.e. national parks and game reserves. It is therefore, critical to identify and safeguard sites where freshwater crab species threatened in the short term through the rehabilitating (e.g. Tanganda Tea Estate and the Rainforest Alliance partnership), strengthening and expanding systems of protected areas (i.e. national parks), private reserves, and other conservation sites containing critical habitats, must be the top priority so as to maximize the return from conservation investments [52].

In conclusion, this is the first study to investigate the distribution and conservation status of freshwater crabs of the genus *Potamonautes* in Eastern Highlands of Zimbabwe. Cumberlidge and Daniels [13], highlighted that the freshwater crab conservation action plans and strategies should be primarily aimed at preserving the integrity of habitats while at the same time closely monitoring crab populations. The current study may provide important information and insight towards the development of a freshwater crab conservation action plan and strategy within the region. The restricted distribution range of *P*. *mutareensis* in Zimbabwe coupled with the on-going anthropogenic induced habitat and climate change are a cause for concern for the long-term conservation of freshwater crabs. Future studies should aim at expanding the spatial coverage of the study as large tracts of areas currently remain unexplored.

## Supporting Information

S1 Table Differences in environmental variables for the two crab species. (DOCX)

S2 Table Abundances of *Potamonautes* in the Eastern Highlands, Zimbabwe. (XLSX)

S1 Text Informal open-ended interviews questions. (DOCX)
