# Supplementary material for: Ecological Assessment of Two Species of Potamonautid Freshwater Crabs from the Eastern Highlands of Zimbabwe, with Implications for Their Conservation
Source: PLoS One. 2016 Jan 11;11(1):e0145923. doi: 10.1371/journal.pone.0145923 (PMC4713832; doi:10.1371/journal.pone.0145923)
Supplement: S1 Table — (DOCX) [file pone.0145923.s001.docx]

**S1 Table.** Differences in environmental variables for the two crab species. Abbreviations: DO – dissolved oxygen, TDS – total dissolved solids

| **Parameter** | ***P. mutareensis*** | | | ***P. unispinus*** | | |
| --- | --- | --- | --- | --- | --- | --- |
|  | **min** | **max** | **mean** | **min** | **max** | **mean** |
| Abundances | 7 | 63 | 33.5±13.7 | 5 | 80 | 23.7±16.5 |
| Elevation (m) | 673 | 2137 | 1321±414 | 613 | 1831 | 1155±407 |
| **Physico-chemical** |  |  |  |  |  |  |
| DO (mg L^-1^) | 1.7 | 7.5 | 5.5±3.9 | 4 | 9.3 | 6.5±2.7 |
| pH | 4.4 | 8 | 7.1±0.8 | 6.3 | 8.3 | 7.3±0.6 |
| TDS (ppt) | 4.6 | 82.5 | 30.4±22.1 | 14.8 | 119 | 39.6±25.2 |
| Conductivity (ppm) | 6.5 | 111 | 41.1±29.9 | 13.6 | 167.9 | 52.8±33.8 |
| Salinity (ppm) | 10.5 | 74 | 27.5±17.2 | 15.4 | 92.3 | 35.7±20.3 |
| Temperature (°C) | 16.2 | 25.4 | 20.6±2.3 | 18.5 | 28.6 | 21.4±1.9 |
| Water depth (m) | 0.1 | 1.6 | 0.5±0.3 | 0.1 | 1.2 | 0.5±0.3 |
| Channel width (m) | 1 | 15 | 3.5±2.4 | 1 | 20 | 6.4±4.8 |
| **Nutrients** |  |  |  |  |  |  |
| Phosphates (mg L^-1^) | 0.1 | 3.5 | 1.1±0.7 | 0.2 | 4.5 | 1.4±1 |
| Ammonium (mg L^-1^) | 0 | 1.8 | 0.4±0.4 | 0 | 2.2 | 0.6±0.7 |
| **Substratum** |  |  |  |  |  |  |
| Clay/silt | 0 | 0.9 | 0.2±0.2 | 0 | 0.9 | 0.2±0.2 |
| Sand | 0 | 0.8 | 0.3±0.3 | 0.1 | 0.8 | 0.4±0.2 |
| Pebbles/cobbles | 0 | 0.9 | 0.4±0.3 | 0.1 | 0.8 | 0.4±0.3 |
| Bedrock | 0 | 0.8 | 0.1±0.2 | 0 | 0.6 | 0.04±0.1 |
| **Vegetation** |  |  |  |  |  |  |
| Macrophyte cover | 0 | 0.7 | 0.3±0.2 | 0.1 | 0.8 | 0.4±0.2 |
| Detritus composition | 0.1 | 0.9 | 0.5±0.2 | 0.1 | 0.8 | 0.4±0.3 |
