# Supplementary material for: Ecological Assessment of Two Species of Potamonautid Freshwater Crabs from the Eastern Highlands of Zimbabwe, with Implications for Their Conservation
Source: PLoS One. 2016 Jan 11;11(1):e0145923. doi: 10.1371/journal.pone.0145923 (PMC4713832; doi:10.1371/journal.pone.0145923)
Supplement: S1 Text — (DOCX) [file pone.0145923.s003.docx]

**S1 Interview questions.** Informal open-ended interviews questions

1. Are you aware of any crabs in your area?
2. What ecological role do you think crabs play?
3. Do you consume crabs as a food?
4. Do you harvest them to sell?
5. For how long have you harvested crabs?
6. How often do you harvest crabs for food or for sale?
7. Are there any specific time when you harvest or do you harvest all year round?
8. Are there any reasons why you harvest in those times?
9. Have you observed any changes in the availability of crabs?
10. Are there any specific locations where you tend to find crabs for harvesting in larger quantities?
11. If so, what do you think accounts for those changes and differences?
12. Are there any measures that you take to maintain the availability of crabs for harvesting?
13. Do you feel it is worthwhile for you to take these measures in order to maintain crab yields?
14. Would you encourage other community members and leaders to take measures to maintain the availability of crabs?
15. What income generating activity are you primarily involved in during the year?
